# Neuroprotective Effect of DAHP via Antiapoptosis in Cerebral Ischemia

**DOI:** 10.1155/2018/5050469

**Published:** 2018-06-19

**Authors:** Yanhua Qin, Weiming Hu, Yang Yang, Zhiying Hu, Weiyun Li, Marong Fang

**Affiliations:** ^1^Department of Psychiatry, Sir Run Run Shaw Hospital, Zhejiang University School of Medicine, Hangzhou, China; ^2^Department of Psychiatry, The Third Hospital of Quzhou, Quzhou, China; ^3^Institute of Neuroscience, Zhejiang University School of Medicine, Hangzhou, China; ^4^Department of Obstetrics and Gynecology, Hangzhou Red Cross Hospital, Hangzhou, China

## Abstract

Aberrant production of nitric oxide following inducible nitric oxide synthase (iNOS) expression has been implicated in cell death and contributes to ischemic brain injury. Tetrahydrobiopterin (BH4) is an essential cofactor of NOS activity. Herein, we evaluated antiapoptotic and anti-inflammatory effects of diamino-6-hydroxypyrimidine (DAHP), a guanosine 5′-triphosphate cyclohydrolase 1 (GTPCH1) inhibitor on focal cerebral ischemia-reperfusion injury by middle cerebral artery occlusion and reperfusion (MCAO) and investigated the underlying mechanism. Sprague-Dawley rats were divided into five groups. Experimental groups were subjected to 1.5 h transient MCAO. T2-weighted imaging was performed to evaluate brain edema lesions in the stroke rats. Infarct volume was estimated by 2,3,5-triphenyltetrazolium chloride (TTC) staining after 24 h reperfusion. Western blotting and immunohistochemistry were performed to detect iNOS, caspase-3, Bcl-2, COX-2, and TNF-*α* protein expressions. Apoptosis was determined by TUNEL staining. T2 hyperintensity changes were observed in primary ischemic region. DAHP pretreatment significantly suppressed iNOS overexpression, caspase-3, and TNF-*α*. There was also attenuation of neuronal apoptosis with decrement in proteins Bcl-2 and COX-2 expressions. On the basis of our results, we hypothesize DAHP to have a neuroprotective function against focal cerebral ischemia and might attenuate brain injury by decreasing reactive oxygen species (ROS) production, subsequently inhibiting apoptosis.

## 1. Introduction

Localized reduction of brain blood flow can result in cerebral ischemia, and prolonged ischemia causes brain infarction, which in turn could result in death [1]. There are two kinds of stroke—ischemic strokes, which are caused by blood clots, and hemorrhagic strokes. Statistics pertaining to demise of individuals following ischemic cardiovascular stroke in 2008 was 7.3 million, with 6.2 million individuals dying from stroke or other cerebrovascular disease in 2008. Stroke or cerebrovascular diseases account for about 4.9% (about 450000 people) of total deaths in low-income countries, thus making it the sixth most common cause of death [2]. Similarly, in 2011, stroke or cerebrovascular diseases accounted for 12.8% (4.91 million people) and 8.9% (790000 people) of total deaths in middle-income and developed countries, respectively, thus making it the second common cause of death in these countries [3].

Thrombolytic therapy, which is deliquescing of blood clot, has been reported to be the optimum therapy for ischemic stroke. Effectiveness of this therapy lies in it being executed within the first 6 hours (the golden hours) following onset of stroke symptoms. Nevertheless, many patients miss the golden hours and are treated with antiplatelet aggregation drugs such as aspirin. Antiplatelet aggregations drugs have the tendency of decreasing risk of reattack; however, they neither allay nor reverse damage caused by stroke [4].

Multitudinous researches have been done on inflammatory response and apoptosis caused by strokes in humans. Several different cytokines are implicated in inflammatory process. These cytokines once stimulated could either upregulate or downregulate substrates, resulting in excessive production of ROS such as hydroxyl radicals (OH^−^), superoxide anions (O_2_^(−)^), and hydrogen peroxide (H_2_O_2_) as well as reactive nitrogen species (RNS) such as nitric oxide (NO) and peroxynitrite (OONO^−^) [5]. Aberrant production of ROS and RNS further results in cell and tissue injury. Some of the cytokines implicated in inflammation include caspase-3 (C3), inducible nitric oxide syntheses (iNOS), and tumor necrosis factor *α* (TNF-*α*) [6].

Production of RNS has been associated with nitric oxide syntheses (NOS). Both endothelial NOS (eNOS) and neuronal NOS (nNOS) are expressed constitutively whereas iNOS expression is induced by proinflammatory cytokines such as TNF-*α*. Increased NOS activity has been reported to be accompanied by GTPCH 1 activation and tetrahydrobiopterin (BH4) formation [7]. BH4 is a requisite for NOS to produce nitric oxide (NO); thus, decreased BH4 curtails production of NO and oxidative stress [8]. BH4 is susceptible to oxidation by ROS, and inflammatory cytokines can increase BH4 synthesis. However, inflammation can also oxidate BH4 due to increasing production of ROS, which in turn forms dihydrobiopterin (BH2) and ultimately biopterin (B) [9]. BH4 synthesis mainly occurs from substrate GTP via a de novo pathway comprised of three steps, with GTP cyclohydrolase 1 (GTPCH 1) being the rate-limiting enzyme under normal conditions. Inhibition of BH4 synthesis via diamino-6-hydroxypyrimidine (DAHP; 0.5 g/kg IP) [10], the inhibitor of GTPCH 1, was reported to abate production of BH4 levels, iNOS activity, and ONOO^−^ levels as well as cerebral infraction [8]. Though hampering of endogenous brain BH4 rate-limiting enzyme GTPCH 1 with DAHP reduces cerebral infarction via iNOS activity inhibition, its neuronal protective mechanism remains elusive. In view of this, our study was aimed at elucidating the mechanistic neuronal protection by DAHP, subsequently providing a theoretical basis in the development of clinical drugs for ischemia stroke treatment.

## 2. Materials and Method

### 2.1. Animal Preparation

All animal procedures and protocols were performed in accordance with the Guide for the Care and Use of Laboratory Animals (NIH publication, 85-23, revised 1996) and were reviewed and approved by the Ethics Committee for the Use of Experimental Animals in Zhejiang University. All male adult Sprague-Dawley (SD) rats (Zhejiang University Laboratory Animal Breeding and Research Center, Hangzhou, China) were housed at a constant temperature (25°C) and humidity (50–60%) under a regular 12 h light/dark schedule, with free access to food and water before and after ischemic induction. Seventy-five adult male Sprague-Dawley rats (weighing 250–300 g) were randomly assigned to 5 groups: normal control (*n* = 15), sham group (*n* = 15), middle cerebral artery occlusion (MCAO, *n* = 15), MCAO with DAHP treatment (0.5 g/kg IP, dissolved in 0.5 ml dimethyl sulfoxide (DMSO)) 12 hours before ischemia (*n* = 15), and MCAO with vehicle (0.5 ml DMSO IP) 12 hours before ischemia (*n* = 15). Both DMSO and DAHP were purchased from Sigma-Aldrich Co., USA.

### 2.2. Induction of Cerebral Ischemic Reperfusion Injury

Methods of cerebral ischemic reperfusion injury were from previous research [11]. Rats were anesthetized with chloral hydrate (400 mg/kg) intraperitoneally. Under anesthesia, right common carotid arteries (CCA), external carotid artery (ECA), and internal carotid artery (ICA) were exposed via a ventral midline incision of the neck. After carefully isolating CCA and separating from the adjacent vagus nerve, CCA and ECA were ligatured with two surgical wires. An incision was made distal to the CCA ligation. Then, a piece of 26/0 monofilament nylon suture with its tip slightly rounded by heat was inserted through the right CCA incision and advanced a distance of 18 mm from the carotid bifurcation toward the origin of right middle cerebral artery, thus occluding blood flow to cortex and striatum. After 150 minutes, reperfusion was accomplished by careful withdrawal of nylon suture; CCA was ligatured distal to the incision, with muscular tissue and skin sewed up. Finally, animals were put into their respective cages. MRI was employed to ascertain successfulness of model. 24 hours after surgery, rats were anesthetized again, and brains were rapidly removed for TTC stain, TUNEL assay, and immunohistochemistry.

### 2.3. MRI Scanning

Rat brains were scanned in a 1.5-Tesla (T) MRI animal scanner (MAGNETOM Trio with TIM system, Siemens, Erlangen, Germany) with the MRI parameters setting at TE = 92 ms, TR = 3620 ms, FOV = 8 × 8 cm^2^, M = 256 × 256, NA = 2, thickness = 2 mm, and gap = 0 mm.

### 2.4. TTC for Infarct Volume Assessment

Each brain was cut at 2 mm intervals from the frontal pole using a rat brain matrix. Sections were stained with 2% 2,3,5-triphenyltetrazolium chloride (TTC, Sigma-Aldrich Co., USA) at 37°C for 10 minutes, fixed in 4% paraformaldehyde overnight, and photographed.

### 2.5. Apoptosis Assay

Terminal deoxynucleotidyl transferase-mediated UTP end labeling (TUNEL) assay was conducted by using a TUNEL detection kit following the manufacturer's instruction (Roche, Mannheim, Germany). Extent of TUNEL was normalized to total nuclear content using 4′,6-diamidino-2-phenylindole (DAPI) staining. Sections were treated with 5 *μ*g/ml proteinase K for 2 minutes at room temperature and rewashed with PBS. Then, sections were treated with a TUNEL reaction mixture at 37°C for 1 hour. DAPI (Sigma-Aldrich Co., USA) was diluted in PBS. For DAPI staining, a 500x stock solution was diluted to 10x PBS; 100 *μ*l of this 10x DAPI solution was then added into each section to achieve a final DAPI loading solution of 10 *μ*M. 10 minutes after, unincorporated DAPI solution was removed and 500 *μ*l of fresh PBS was added to sections. TUNEL-stained sections stained with DAPI were visualized with a confocal laser scanning inverted microscope (Meta Zeiss LSM 510). Positive cells were counted in 5 random fields per section at 200x magnification.

### 2.6. Immunohistochemistry

24 hours following reperfusion, animals were deeply anesthetized with 4% chloral hydrate and then perfused transcardially with 500 ml of 0.9% saline, followed by 4% paraformaldehyde in 0.01 M PBS. Brains were removed, postfixed in a 4% paraformaldehyde solution, and transferred to a 30% sucrose solution prior to processing for immunostaining. Brains were then frozen and sectioned coronally. Sections were incubated for 15 minutes with 3% hydrogen peroxide in methanol to block endogenous peroxidases and then blocked for 1 hour with 0.3% Triton X-100/10% normal goat serum in 0.01 M PBS. Then, sections were incubated with primary antibody anti-iNOS, TNF-*α*, COX-2, caspase-3, and Bcl-2 (rabbit, 1 : 200, Thermo Fisher Scientific, Waltham, MA) overnight at 4°C. After a thorough washing in PBS, sections were incubated with HRP-conjugated goat anti-rabbit IgG (1 : 200; Santa Cruz Biotechnology, CA) for 1 hour and then visualized with 3,3′-diaminobenzidine (DAB, Sigma-Aldrich Co., USA). Positive cells in each section were counted at 200x magnification.

### 2.7. Western Blot Analysis

Total protein was extracted from right brain tissues of each group. Protein concentration of extract was determined by Bio-Rad DC protein assay. Equal amounts of tissue extracts containing 30 *μ*g of total protein were boiled for 5 minutes. Denatured proteins were separated by SDS-PAGE and transferred to polyvinylidene difluoride (PVDF) membrane in a Bio-Rad Trans-Blot apparatus. Membranes were blocked with TBST containing 5% nonfat milk for 2 hours at room temperature and then incubated with primary rabbit polyclonal anti-COX-2 (1 : 300), anti-Bcl-2 (1 : 500), anti-iNOS (1 : 300), and anti-caspase-3 (1 : 300) on a platform shaker overnight at 4°C. Equivalent loading of protein across all test lanes was confirmed by subsequent *β*-actin immunoblots (anti-*β*-actin 1 : 200; Biomeda) after incubation with horseradish peroxidase-conjugated goat anti-rabbit antibody (1 : 3000; Jackson ImmunoResearch Laboratories, USA) for 1 hour at room temperature and ECL detection.

### 2.8. Statistical Analysis

Data are presented as mean ± standard error of mean (SEM). One-way analysis of variance (ANOVA) was used to determine statistical significance with SPSS 16.0 program. Comparison among groups was performed by ANOVA followed by LSD-Fisher post hoc test. A *P* value of <0.05 was considered statistically significant. All statistical analysis and graphs were performed or generated with GraphPad Prism Version 4.0 (GraphPad Prism Software Inc., CA).

## 3. Results and Discussion

### 3.1. MRI Assessment of MCAO Model

**Figure 1:**
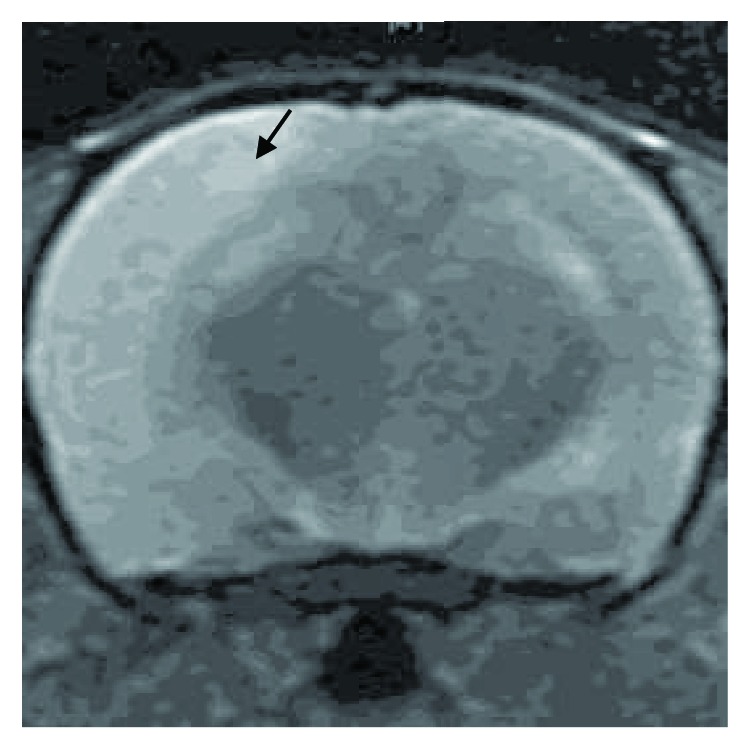
Infarct region performed T2 hyperintensity changes. T2-weighted magnetic resonance image revealed an abnormally increased T2 signal in the primary ischemic region (arrow).

MRI is considered to be the most sensitive tool in evaluation of brain edema lesions in stroke victims. After 24 hours of surgery, T2-weighted imaging was performed in stroke rats and T2 hyperintensity changes were observed in primary ischemic region. Representative image is shown in Figure 1. Infarct lesion was observed 24 hours after reperfusion in our MCAO model.

### 3.2. DAHP Curtails Infarct Volume

**Figure 2:**
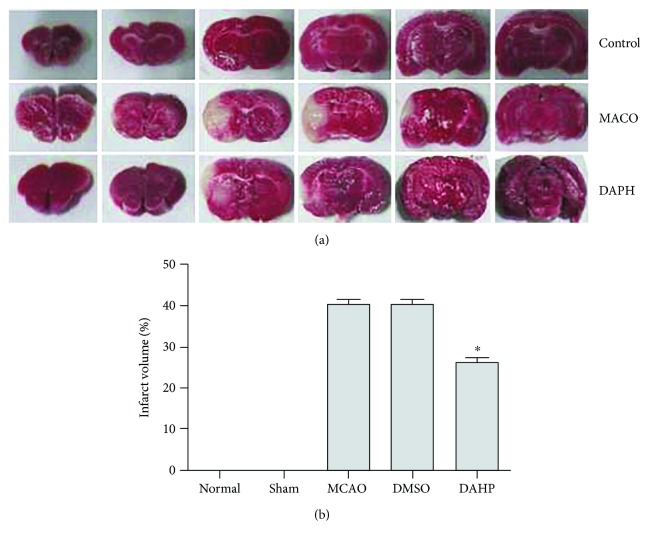
(a) Unstained areas (negative TTC stains) were defined as infarct regions, while normal regions appeared red. (b) Bar graph represents the infarct volume from TTC staining in various groups (mean ± SEM, *n* = 6, ^∗^*P* < 0.05 versus the DAHP-treated group). Infarct volume markedly decreased in the DAHP-treated group, compared with the MCAO group (*n* = 6, ^∗^*P* < 0.05 versus the DAHP-treated group).

In evaluating DAHP neuroprotectiveness, infarct volume was measured at 24 hours following reperfusion. Brains of rats were stained with 2% TTC to obtain infarct volume. Unstained areas (negative TTC stains) that appeared white were defined as infarct regions whereas normal regions appeared red (Figure 2(a)). Infarct volume (mm^3^) was calculated as 2 mm (thickness of the slice) × sum of the infarction area (mm^2^) in all slices using computerized planimetry (PC-based image tool software). As evidenced in Figure 2(b), infarct volume of the DAHP group was significantly decreased in comparison to the MCAO group (^∗^*P* < 0.05, versus DAHP-treated group). No cerebral infarction was observed in the sham group. Infarct volume suggests DAHP plays a neuroprotective role in brain infarction.

### 3.3. DAHP Mitigates Neuronal Apoptosis

**Figure 3:**
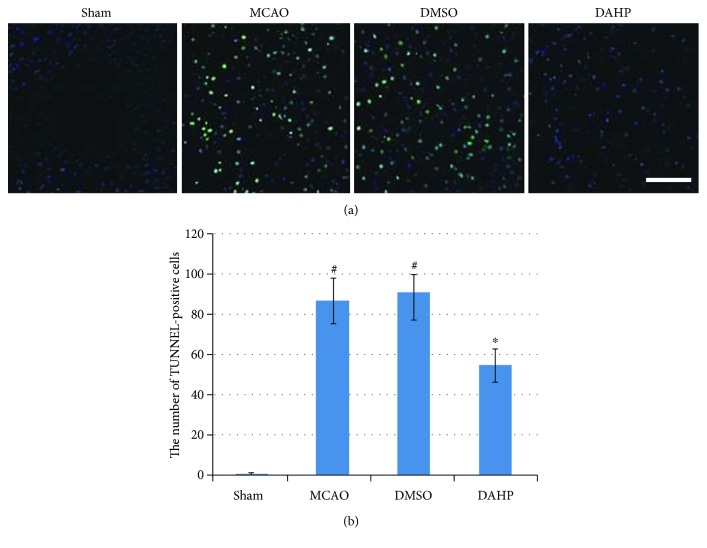
(a) Representative feature of TUNEL-positive cells in the ischemia area. (b) Bar graph reflected the TUNEL-positive staining score in each group (mean ± SEM, *n* = 6, ^∗^*P* < 0.05 versus the DAHP-treated group). Ischemic reperfusion caused much neuron apoptosis; this can be demonstrated by the MCAO group (^#^*P* < 0.05 versus the sham group) with reduced neuron apoptosis compared with the MCAO group (*n* = 6, ^∗^*P* < 0.05), and DMSO failed to attenuate apoptosis.

TUNEL-positive cell represents apoptotic cell. As illustrated in Figure 3(a), at 24 hours following reperfusion, number of apoptotic cells in penumbral area and infarct zone was observed. TUNEL method can identify DNA fragmentation of apoptotic cells. As depicted in Figure 3(b), in comparison to sham rats, number of TUNEL-positive cells was significantly increased in both MCAO and DMSO groups (^#^*P* < 0.05, versus sham group). In the DAHP-treated group, number of TUNEL-positive cells was significantly decreased relative to both MCAO and DMSO groups (^∗^*P* < 0.05, versus DAHP-treated group). These results suggest DAHP could effectively obviate apoptotic cells following onset of stroke.

### 3.4. Immunohistochemistry

**Figure 4:**
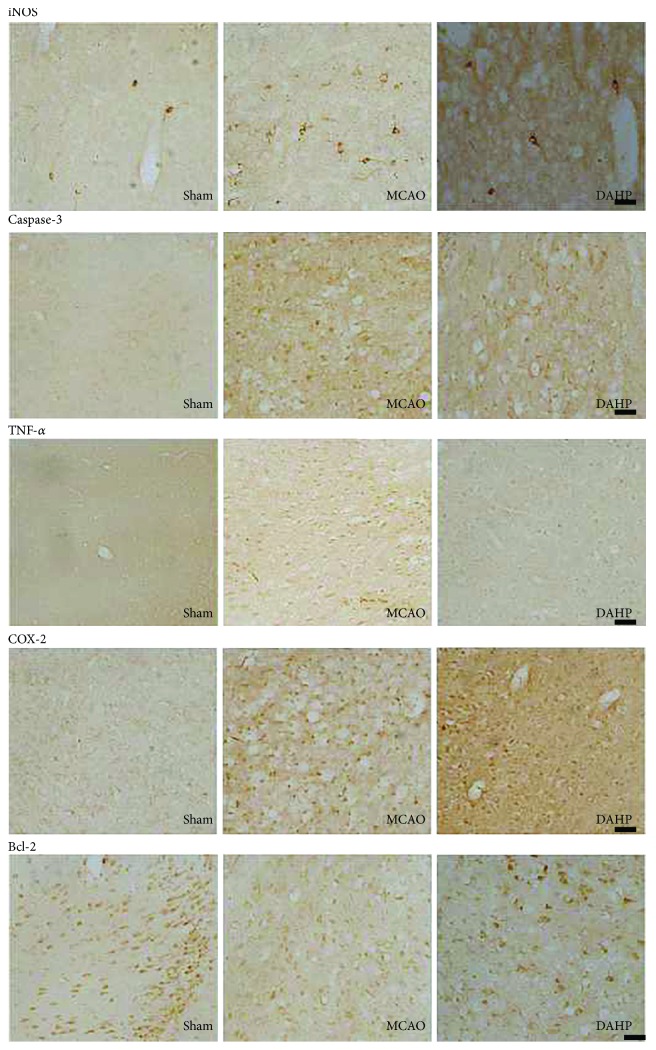
Immunohistochemistry staining shown in penumbral and stroke areas at 24 h after reperfusion. DAHP downregulated the expression levels of caspase-3, TNF-*α*, and iNOS, but upregulated the levels of Bcl-2 and COX-2, when compared with the MCAO group.

**Figure 5:**
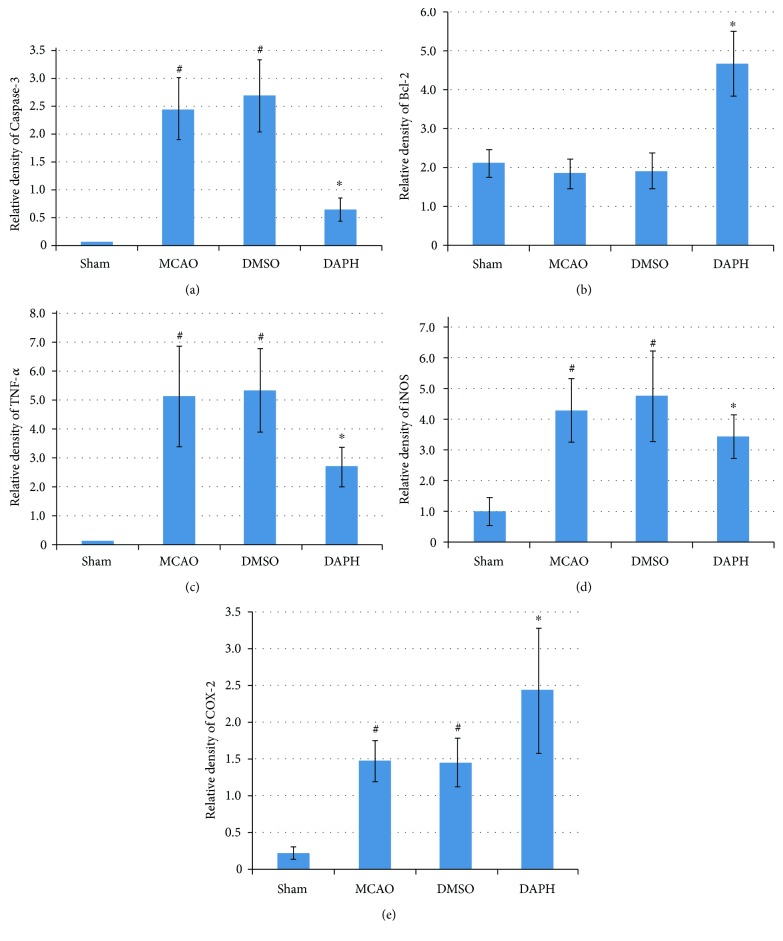
Expression levels for some inflammation cytokines in the rat cortex in penumbral and stroke areas were quantified by IOD with Image Pro Plus 6.0 software. (a) DAHP downregulated the caspase-3 expression (^∗^*P* < 0.05, versus the DAHP-treated group). Ischemic reperfusion induced a substantial caspase-3 expression (^#^*P* < 0.05, versus the sham group), which was significantly attenuated by DAHP but not vehicle treatment and MCAO groups (*n* = 4, ^∗^*P* < 0.05, versus the DAHP-treated group). (b) DAHP increased Bcl-2 expression. The total Bcl-2 expression was significantly increased 24 h after ischemic reperfusion in the DAHP group (*n* = 4, ^∗^*P* < 0.05, versus the DAHP-treated group), relative to MCAO or vehicle treatment. (c) DAHP reduced TNF-*α* expression. Ischemic reperfusion induced a substantial increase in TNF-*α* expression (^#^*P* < 0.05, versus the sham group), which reduced in the DAHP-treated group (*n* = 3, ^∗^*P* < 0.05, versus the DAHP-treated group). (d) DAHP attenuated iNOS expression. Ischemic reperfusion induced a substantial increase in iNOS expression (^#^*P* < 0.05, versus the sham group), which was significantly attenuated by DAHP (*n* = 4, ^∗^*P* < 0.05, versus the DAHP-treated group). (e) DAHP induced COX-2 expression; DAHP increased COX-2 expression (*n* = 4, ^∗^*P* < 0.05, versus the DAHP-treated group).

Inflammatory cytokine expression in an MCAO rat model was detected via immunohistochemistry analysis in penumbral and stroke area at 24 hours after reperfusion. Positive staining cells were brown in color (Figure 4). Bcl-2, TNF-*α*, caspase-3, cyclooxygenase-2 (COX-2), and iNOS expressions in rat cortex were delineated, and the integral optical density (IOD) was measured with Image-Pro Plus 6.0 software. In comparison to MCAO group, DAHP suppressed the expressions of apoptotic cytokines, caspase-3, TNF-*α*, and iNOS (^∗^*P* < 0.05 versus DAHP-treated group); however, expressions of antiapoptotic cytokines, Bcl-2, and COX-2 were stimulated (^∗^*P* < 0.05 versus the DAHP-treated group) (Figure 5). Cytokine expressions between MCAO and DMSO groups were not statistically different (*P* > 0.05).

### 3.5. Western Blotting

**Figure 6:**
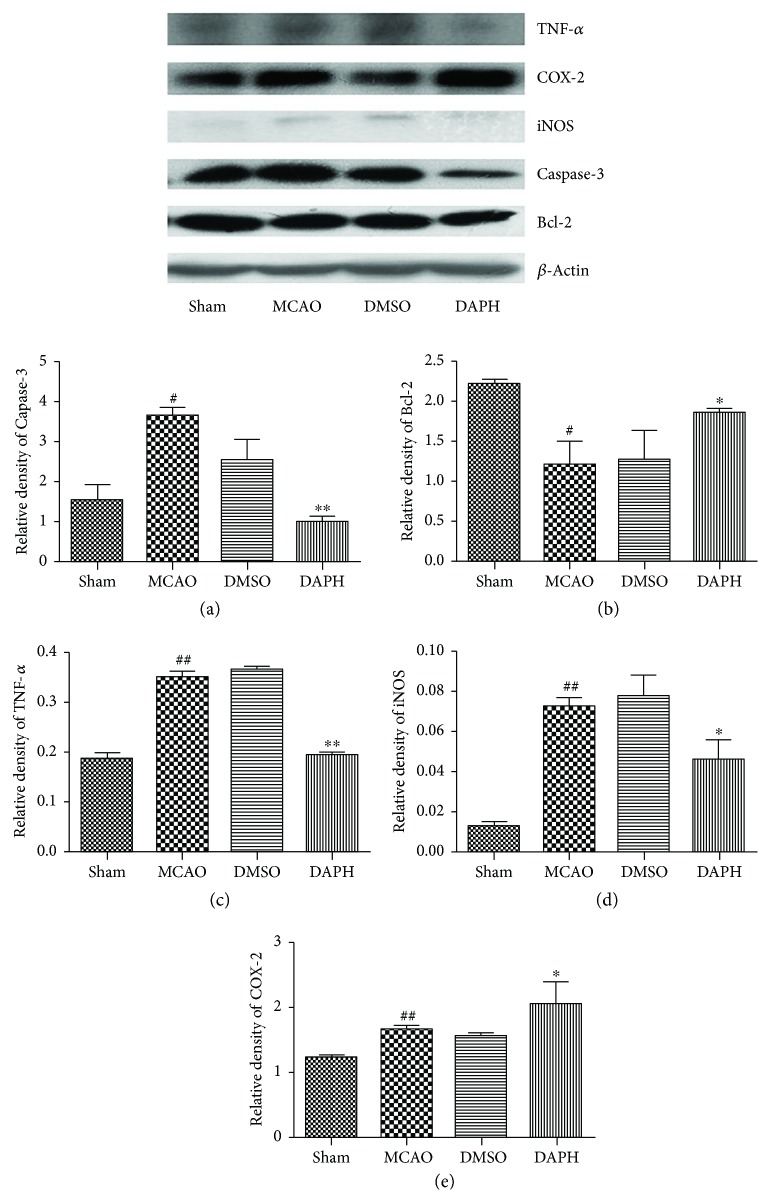
Protein expression levels for inflammation cytokines in the rat cortex in penumbral and stroke areas were quantified by Western blotting. (a, c, d) DAHP downregulated caspase-3, TNF-*α*, and iNOS protein expression. Ischemic reperfusion induced a substantial caspase-3, TNF-*α*, and iNOS expression in the vehicle treatment and MCAO groups (^#^*P* < 0.05, versus the sham group), which was significantly attenuated by DAHP (^∗^*P* < 0.05 and ^∗∗^*P* < 0.05, versus the DAHP-treated group). (b) DAHP increased Bcl-2 expression (^∗^*P* < 0.05, versus the DAHP-treated group). Total Bcl-2 expression was significantly increased 24 h after ischemic reperfusion in the DAHP group, relative to MCAO or vehicle treatment. (e) DAHP induced COX-2 protein expression. Ischemic reperfusion induced COX-2 expression in the MCAO- and DMSO-treated groups, but COX-2 expression was more upregulated in the DAHP group than in the MCAO and DMSO groups (^∗^*P* < 0.05, versus the DAHP-treated group). ^##^*P* < 0.05, versus the sham group (c–e).

Western blotting was used to quantify cytokine protein expression in MCAO rat models. In comparison to MCAO group, expressions of apoptotic cytokines, caspase-3, TNF-*α*, and iNOS were suppressed by DAHP (^∗^*P* < 0.05 and ^∗∗^*P* < 0.05 versus DAHP-treated group) (Figures 6(a), 6(c), and 6(d)). However, antiapoptotic cytokine expressions of Bcl-2 and COX-2 were stimulated (^∗^*P* < 0.05 versus the DAHP-treated group) (Figures 6(b) and 6(e)). Cytokine expressions between MCAO and DMSO groups were not statistically different.

### 3.6. Discussion

We evidence neuroprotectiveness of DAHP in an MCAO rat model and report our findings following Western blot and immunohistochemical analyses as well as TUNEL staining.

Stroke is a global health problem and the leading cause of adult disability in the United States. Thrombolytic therapy is currently the most effective treatment. However, there is a concomitant increased risk of bleeding coupled with irreversibility of damage caused. In view of this, exploration of alternative therapeutics for ischemia stroke in clinical setting is paramount. In our study, we occluded blood flow to the brain and subsequently cut off immediate oxygen supply to neurons. In our study, we occluded blood flow to the brain and subsequently cut off immediate oxygen supply to neurons, leading to death of cells (Figure 2(a)).

In this study, iNOS expression was suppressed via DAHP which is possibly due to inhibition of GTPCH 1 activity to decrease BH4 synthesis. This was in contrast with previous studies that reported iNOS protein level to be not downregulated but with a reduced activity [8]. Accumulated toxic products, ROS and RNS, can tip the balance between life and death [12]. RNS and ROS can break DNA and induce lipid peroxidation, protein nitrosylation of cell membrane and organelle damage of cell and tissue. Thus, downregulated iNOS by DAHP resulted in less RNS and ROS, which in turn decreased neuronal injury [13].

Apoptotic cell death pathways implicated in brain injury following cerebral ischemia have been elucidated [10]. Apoptosis is a form of cell death and is composed of two pathways—intrinsic pathway (mitochondrial pathway) and extrinsic pathway. Furthermore, the central role of apoptosis is ascribed to its elimination of dying cells so as to maintain normal development [14]. Excessive apoptosis, however, is presented in some pathological conditions such as stroke intracerebral hemorrhage, cancer, autoimmune disorders, and heart disease [15, 16]. Various reactive oxygen and nitrogen species (ROS/RNS) regulate apoptosis.

ROS and RNS have been demonstrated to have both pro- and antiapoptotic roles depending on a variety of factors such as type of cells involved, redox state of cell, as well as flux and dose of NO. Also, RNS and ROS can trigger inflammation via disparate cytokines such as TNF-*α* and caspase-3. As a proinflammatory cytokine, TNF-*α* has been reported to activate apoptosis through either intrinsic or extrinsic pathway via activation of caspase-3 by caspase-8 ([17]). Caspase-3 is a member of caspase family, which are essential initiators and executioners in apoptosis; caspase-3 acts as an executioner [18]. Caspase-3 has been reported to be overexpressed in myocardial infarction [19]. We found caspase-3 to be excessively expressed in infarct lesions of MCAO group. In essence, apoptosis plays an essential role in stroke pathology, while DAHP treatment markedly decreased caspase-3 protein in our study; thus, DAHP neuroprotective effects might arise following suppression of proapoptotic factors. Besides, members of Bcl-2 family, the major regulators of mitochondrial apoptotic pathway, are associated with pro- and antiapoptosis [20]. Antiapoptotic effect of Bcl-2 protein has been reported to be so because of Bcl-2 possessing four BH domains [5]. Additionally, Bcl-2 can suppress the level of free radicals or regulate cellular antioxidants to impede apoptosis [21]. Decreased infarct volume and upregulated Bcl-2 in our DAHP group implies DAHP proffered protection to neurons from death, possibly via antiapoptosis.

COX-2's role in cerebral infarction is presently ambiguous. COX-2 activation protects cardiomyocytes against oxidative stress [22]. Inhibition of COX-2 exacerbates inflammation and hippocampal neuronal death induced by seizures [23]. Hippocampal neuronal cells are attenuated in COX-2-deficient mouse death following transient forebrain ischemia [23, 24]. COX-2 is a major mediator implicated in inflammation and ischemic stroke [25]. In our study, COX-2 expression was upregulated more in DAHP group than in MCAO and DMSO groups. Thus, we hypothesize COX-2 might play a protective role in brain injury. We believe that following occlusion of blood flow to the brain, protective effect of COX-2 was set in motion against neuronal death, and with the assistance of DAHP, its expression and protective effect was enhanced, subsequently culminating in curtailed inflammation and RNS.

## 4. Conclusions

In summation, DAHP attenuates brain injury in a rat model through iNOS signaling pathway as well as apoptosis.

## Data Availability

The data used to support the findings of this study are available from the corresponding author upon request.
